# Supplementary material for: HIVprotI: an integrated web based platform for prediction and design of HIV proteins inhibitors
Source: J Cheminform. 2018 Mar 9;10:12. doi: 10.1186/s13321-018-0266-y (PMC5845081; doi:10.1186/s13321-018-0266-y)
Supplement: Supplementary file 2 — Additional file 2. Source code of HIVProtI web server. [file 13321_2018_266_MOESM2_ESM.zip › HIVprotI_Source-code/hivprotI/weka-3-6-6/doc/weka/core/converters/Saver.html]

Saver


---


|  |  |  |
| --- | --- | --- |
| **Overview** \| **Package** \| **Class** \| **Tree** \| **Deprecated** \| **Index** \| **Help** \| **Weka's home** | |  |
| **PREV CLASS**   **NEXT CLASS** | **FRAMES**    **NO FRAMES**     **All Classes** |  |
| SUMMARY: NESTED \| FIELD \| CONSTR \| METHOD | DETAIL: FIELD \| CONSTR \| METHOD |  |


---


## weka.core.converters Interface Saver

**All Superinterfaces:**: RevisionHandler, java.io.Serializable

**All Known Implementing Classes:**: AbstractFileSaver, AbstractSaver, ArffSaver, C45Saver, CSVSaver, DatabaseSaver, LibSVMSaver, SerializedInstancesSaver, SVMLightSaver, XRFFSaver

---

``` public interface Saver extends java.io.Serializable, RevisionHandler ```

Interface to something that can save Instances to an output destination in some
format.

**Version:**
:   $Revision: 1.4 $

**Author:**
:   Mark Hall (mhall@cs.waikato.ac.nz), Stefan Mutter (mutter@cs.waikato.ac.nz)

---

| **Field Summary** | |
| --- | --- |
| `static int` | `BATCH` |
| `static int` | `INCREMENTAL` |
| `static int` | `NONE`             The retrieval modes |


| **Method Summary** | |
| --- | --- |
| `java.lang.String` | `filePrefix()`             Gets the file prefix This method is used in the KnowledgeFlow GUI. |
| `java.lang.String` | `getFileExtension()`             Gets the file extension |
| `int` | `getWriteMode()`             Gets the write mode |
| `java.lang.String` | `retrieveDir()`             Gets the driectory of the output file This method is used in the KnowledgeFlow GUI. |
| `void` | `setDestination(java.io.File file)`             Resets the Saver object and sets the destination to be the supplied File object. |
| `void` | `setDestination(java.io.OutputStream output)`             Resets the Saver object and sets the destination to be the supplied InputStream. |
| `void` | `setDir(java.lang.String dir)`             Sets the directory of the output file. |
| `void` | `setDirAndPrefix(java.lang.String relationName, java.lang.String add)`             Sets the file prefix and the directory. |
| `void` | `setFile(java.io.File file)`             Sets the output file |
| `void` | `setFilePrefix(java.lang.String prefix)`             Sets the file prefix. |
| `void` | `setInstances(Instances instances)`             Sets the instances to be saved |
| `void` | `setRetrieval(int mode)`             Sets the retrieval mode |
| `void` | `writeBatch()`             Writes to a destination in batch mode |
| `void` | `writeIncremental(Instance inst)`             Writes to a destination in incremental mode. |

**Methods inherited from interface weka.core.RevisionHandler**

`getRevision`

**Field Detail**

### NONE

```
static final int NONE
```

:   The retrieval modes

    **See Also:**: Constant Field Values

---


### BATCH

```
static final int BATCH
```

**See Also:**: Constant Field Values

---


### INCREMENTAL

```
static final int INCREMENTAL
```

**See Also:**: Constant Field Values


**Method Detail**

### setDestination

```
void setDestination(java.io.File file)
                    throws java.io.IOException
```

:   Resets the Saver object and sets the destination to be
    the supplied File object.

    :   **Parameters:**: `file` - the File **Throws:**: `java.io.IOException` - if an error occurs support loading from a File. ``` public_normal_behavior requires: file != null && (* file exists *); modifiable: model_sourceSupplied, model_structureDetermined; ensures: model_sourceSupplied == true && model_structureDetermined == false; also public_exceptional_behavior requires: file == null || (* file does not exist *); signals: (IOException); ```

---


### setDestination

```
void setDestination(java.io.OutputStream output)
                    throws java.io.IOException
```

:   Resets the Saver object and sets the destination to be
    the supplied InputStream.

    :   **Parameters:**: `output` - the output stream **Throws:**: `java.io.IOException` - if this Loader doesn't support loading from a File.

---


### setRetrieval

```
void setRetrieval(int mode)
```

:   Sets the retrieval mode

    :   **Parameters:**: `mode` - an integer representing a retrieval mode

---


### getFileExtension

```
java.lang.String getFileExtension()
                                  throws java.lang.Exception
```

:   Gets the file extension

    :   **Returns:**: a string conatining the file extension (including the '.') **Throws:**: `java.lang.Exception` - exception if a Saver not implementing FileSourcedConverter is used.

---


### setFile

```
void setFile(java.io.File file)
             throws java.io.IOException
```

:   Sets the output file

    :   **Parameters:**: `file` - the output file **Throws:**: `java.io.IOException` - exception if new output file cannot be set

---


### setFilePrefix

```
void setFilePrefix(java.lang.String prefix)
                   throws java.lang.Exception
```

:   Sets the file prefix.
    This method is used in the KnowledgeFlow GUI.

    :   **Parameters:**: `prefix` - the prefix of the file name **Throws:**: `java.lang.Exception` - exception if a Saver not implementing FileSourcedConverter is used.

---


### filePrefix

```
java.lang.String filePrefix()
                            throws java.lang.Exception
```

:   Gets the file prefix
    This method is used in the KnowledgeFlow GUI.

    :   **Returns:**: the prefix of the file name **Throws:**: `java.lang.Exception` - exception if a Saver not implementing FileSourcedConverter is used.

---


### setDir

```
void setDir(java.lang.String dir)
            throws java.io.IOException
```

:   Sets the directory of the output file.
    This method is used in the KnowledgeFlow GUI.

    :   **Parameters:**: `dir` - a string containing the path and name of the directory **Throws:**: `java.io.IOException` - exception if a Saver not implementing FileSourcedConverter is used.

---


### setDirAndPrefix

```
void setDirAndPrefix(java.lang.String relationName,
                     java.lang.String add)
                     throws java.io.IOException
```

:   Sets the file prefix and the directory.
    This method is used in the KnowledgeFlow GUI.

    :   **Parameters:**: `relationName` - the name of the realtion to be saved: `add` - additional String for the file name **Throws:**: `java.io.IOException` - exception if a Saver not implementing FileSourcedConverter is used.

---


### retrieveDir

```
java.lang.String retrieveDir()
                             throws java.io.IOException
```

:   Gets the driectory of the output file
    This method is used in the KnowledgeFlow GUI.

    :   **Returns:**: the directory as a string **Throws:**: `java.io.IOException` - exception if a Saver not implementing FileSourcedConverter is used.

---


### setInstances

```
void setInstances(Instances instances)
```

:   Sets the instances to be saved

    :   **Parameters:**: `instances` - the instances

---


### writeBatch

```
void writeBatch()
                throws java.io.IOException
```

:   Writes to a destination in batch mode

    :   **Throws:**: `java.io.IOException` - throws exection if writting in batch mode is not possible

---


### writeIncremental

```
void writeIncremental(Instance inst)
                      throws java.io.IOException
```

:   Writes to a destination in incremental mode.
    If the instance is null, the outputfile will be closed.

    :   **Parameters:**: `inst` - the instance to write, if null the output file is closed **Throws:**: `java.io.IOException` - throws exception if incremental writting is not possible

---


### getWriteMode

```
int getWriteMode()
```

:   Gets the write mode

    :   **Returns:**: an integer representing the write mode


---


|  |  |  |
| --- | --- | --- |
| **Overview** \| **Package** \| **Class** \| **Tree** \| **Deprecated** \| **Index** \| **Help** \| **Weka's home** | |  |
| **PREV CLASS**   **NEXT CLASS** | **FRAMES**    **NO FRAMES**     **All Classes** |  |
| SUMMARY: NESTED \| FIELD \| CONSTR \| METHOD | DETAIL: FIELD \| CONSTR \| METHOD |  |


---
